# Emotional climate, feeding practices, and feeding styles: an observational analysis of the dinner meal in Head Start families

**DOI:** 10.1186/1479-5868-8-60

**Published:** 2011-06-10

**Authors:** Sheryl O Hughes, Thomas G Power, Maria A Papaioannou, Matthew B Cross, Theresa A Nicklas, Sharon K Hall, Richard M Shewchuk

**Affiliations:** 1 USDA/ARS Children's Nutrition Research Center, Department of Pediatrics, Baylor College of Medicine, 1100 Bates Street, Houston, TX 77030-2600, USA; 2 Washington State University, Department of Human Development, P.O. Box 644852, Pullman, WA 99164-4852, USA; 3 University of Houston-Clear Lake, Department of Psychology, 2700 Bay Area Boulevard, Box 155, Houston, TX 77058, USA; 4 University of Alabama at Birmingham, Department of Health Services Administration, 560 Webb Building, 1530 3rd Avenue South, Birmingham, AL 35294-3361, USA

## Abstract

**Background:**

A number of studies conducted with ethnically diverse, low-income samples have found that parents with indulgent feeding styles had children with a higher weight status. Indulgent parents are those who are responsive to their child's emotional states but have problems setting appropriate boundaries with their child. Because the processes through which styles impact child weight are poorly understood, the aim of this study was to observe differences in the emotional climate created by parents (including affect, tone of voice, and gestures) and behavioral feeding practices among those reporting different feeding styles on the Caregiver's Feeding Styles Questionnaire. A secondary aim was to examine differences on child weight status across the feeding styles.

**Methods:**

Participants were 177 Head Start families from Houston, Texas (45% African-American; 55% Hispanic). Using an observational approach, the relationship between the observed emotional climate of the meal, behavioral feeding practices, and self-reported parent feeding styles were examined. Mean age of the children was 4.4 years (SD = 0.7) equally distributed across gender. Families were observed on 3 separate dinner occasions. Heights and weight were measured on the parents and children.

**Results:**

Parents with self-reported indulgent feeding styles made fewer demands on their children to eat during dinner and showed lower levels of negative affect and intrusiveness. Surprisingly, these parents also showed higher levels of emotional detachment with their children during dinner. Hispanic boys with indulgent parents had significantly higher BMI *z *scores compared to Hispanic boys in the other three feeding style groups. No other differences were found on child weight status.

**Conclusions:**

Results suggest that the emotional climate created by indulgent parents during dinner and their lack of demands on their children to eat may play an important role in how young children become overweight. Numerous observed emotional climate and behavioral differences were found between the other self-reported feeding styles as well. Results suggest that parents' self-reported feeding styles may be a proxy for the emotional climate of the dinner meal, which may in turn influence the child's eating behaviors and weight status.

## Introduction

A number of studies with low-income minority families have shown that children of parents with an indulgent parenting or feeding style are at the greatest risk for childhood obesity [1-4]. Indulgent parents are those who are responsive to their child's emotional states but have problems setting appropriate boundaries with their child. In three separate cross-sectional studies of ethnically diverse, low-income parents from different geographical areas of the United States (both urban and rural), researchers found that indulgent parents had children with the highest BMI percentiles [1-3]. In a longitudinal study of parenting and low-income Mexican American children's health behaviors, Olvera and Power [4] found that young children of indulgent mothers were most likely to become overweight three years later. Finally, in a self-serving experimental study, Fisher and colleagues [5] found that young children of parents reporting indulgent feeding styles served themselves and consumed larger entrée portions. Despite the numerous studies showing a link between indulgent feeding and child eating behaviors and weight status, the processes through which parenting and feeding styles impact these child outcomes are not well understood. Indulgent parents are likely to differ from other parents in both the feeding practices they use to get children to eat and in the emotional climate they create during mealtime.

According to parenting theory [6,7], an important distinction has been made between parenting practices and the larger context in which these behaviors are expressed (defined as styles). Styles have the broadest influence on a child's behavior because they create the *emotional climate *within which practices can be accepted or rejected by the child [8]. Styles are considered an emotional context rather than a compilation of specific parenting practices. In contrast, parenting practices are goal-directed behaviors used by parents to get children to do something specific (such as eating their food) [8]. The emotional climate of parent-child interactions is determined by the style. The construct of emotional climate has been inferred from styles and is assumed to be specific to particular styles but has not been explicitly measured within that framework. For example, parents with an authoritarian style are characterized as negative, intrusive, and restrictive but the affect, tone of voice, and gestures corresponding to these emotions and behaviors have not been explicitly measured.

Based on dimensions of responsiveness and demandingness, parenting and/or feeding styles are termed as authoritative if the parent is responsive, involved, and makes appropriate demands on the child (high on both dimensions); authoritarian if the parent is demanding and highly directive but unresponsive to the child's individual needs (high demandingness, low responsiveness); indulgent if the parent is warm and accepting but makes few demands on the child (low demandingness, high responsiveness); and uninvolved if the parent exerts little control over the child and lacks involvement (low on both dimensions) [3]. The authoritative style has been associated with the most positive child outcomes including better social competence, mental health, academic achievement, and weight status [7-9]. The authoritative style is thought to be the most effective because 1) the responsivity and involvement characteristic of authoritative parents allow a child to be more receptive to parenting directives and 2) parent child-interactions among authoritative parents engage a child in a dynamic that fosters self-regulation and competence.

Self-regulation is a key construct in parenting style theory with parents influencing the development of child self-regulation in specific ways (see Power, 2004 for a review) [10]. Self-regulated children have parents who show positive versus negative emotion [11-13]; who are accepting and not dismissing of their children's emotional expression [14,15]; and who are not overly controlling in their approach to children's behavior [16,17]. Thus, parents who are negative, controlling, dismissing and/or detached from their child may foster problems with the development of their child's self-regulation. Negative and dismissive/detached parenting likely contributes to poor self-regulation by increasing the level of children's negative arousal in challenging situations, making it difficult for them to learn, practice, and implement effective coping strategies [10]. In the feeding environment, a consistently maladaptive eating situation may influence how much and what children eat during a meal. Children could learn to use food as a comfort tool if parent-child interactions are maladaptive during mealtimes [18,19].

In addition to the emotional climate contributing to the development of child self-regulation in general, certain types of behavioral feeding practices have also been shown to diminish the ability for children to self-regulate food intake. Highly directive/controlling feeding practices have been linked to lower self-regulation in eating and higher weight status among children across numerous studies (see Clark, Goyder, Bissell, Blank, & Peters, 2007 and Faith, Scranlon, Birch, Francis, & Sherry, 2004 for reviews) [20,21]. For example, children who were instructed to "clean their plates" were less responsive to energy density cues than children who were taught to focus on internal cues of hunger and fullness [22]. Snoek and colleagues [23] found that low maternal support paired with high levels of psychological control was associated with emotional eating in young adolescents. Such findings are often interpreted to suggest that when parents are highly controlling of their children's food intake, their children focus more on external rather than internal cues to regulate intake, leading to lower levels of self-regulation and greater eating in the absence of hunger [24,25]. To our knowledge, however, the emotional climate of the dinner meal in conjunction with highly directive/controlling behavioral feeding practices believed to diminish self-regulation in children has not been explicitly measured in the home meal.

The primary aim of this study was to *observe *differences in the emotional climate created by parents (including affect and other parent behaviors such as tone of voice and gestures) and specific behavioral feeding practices that may interfere with child self-regulation among those reporting different feeding styles on the Caregiver's Feeding Style Questionnaire (CFSQ) [2]. The CFSQ is a parent-report tool measuring parent feeding styles of low-income children. It was developed based on a parenting style paradigm and classifies parents into one of four feeding styles based on the parenting style framework. Using an observational approach to measure parent emotions and feeding practice behaviors during dinner, the relationships between the observed emotional climate of the meal, observed behavioral feeding practices, and self-reported parent feeding styles were examined in low-income families. Regarding emotional climate, it was hypothesized that parents with an authoritative feeding style would be observed to be more sensitive, parents with an authoritarian feeding style would be observed to be more negative and intrusive, indulgent parents would be observed to be more positive, and uninvolved parents would be observed to be more detached compared to the other feeding styles. Regarding specific behavioral feeding practices, it was hypothesized that parents with high demandingness styles (i.e., authoritarian and authoritative) would show more attempts to influence child eating than parents with low demandingness styles (i.e., indulgent and uninvolved). A secondary aim was to examine differences across the feeding styles on child weight status. Parents with an indulgent feeding style were expected to have children with a higher weight status compared to the other styles of feeding.

## Methods

### Participants

A total of 177 families (45% African-American; 55% Hispanic) were recruited from Head Start centers in the Houston metropolitan area. Data were collected on the recruited families in 2007 and 2008. Head Start is a comprehensive child development program that serves preschool children and their families. The overall goal of the program is to increase school readiness in young children from low-income families. Children attend Head Start during the weekdays from 7 a.m. until 2 p.m. with some Head Start centers providing after school care from 2 p.m. to 5 p.m.

Most parents in this study were employed with 39% working full-time and 22% working part-time (see Table 1). Parents showed a wide range of education, with almost one-third having less than a high school diploma and about one-third having some college. Only 7% had college degrees. Children participating in the study ranged from 3 to 5 years with a mean age of 4.4 years (SD = 0.7) equally distributed across child gender. The mean age of the parents was 32 years (SD = 7.7)

**Table 1:** Characteristics of the Sample (*n *= 177)

Ethnicity (%)	
Hispanic	45.2
African-American	54.8
	
Parent gender (%)	
Female	96.8
Male	3.2
	
Education of parent (%)	
Less than a High School diploma	28.7
High School diploma	26.1
Some college	35.6
College graduate	7.4
Post graduate	1.1
Missing data	1.1
	
Child gender (%)	
Female	48.1
Male	51.9
	
Employment of parent (%)	
Employed part-time	21.8
Employed full-time	38.8
Unemployed	38.3
Missing data	1.1
	
Age, mean in years (SD)	
Parent	32.03 (7.698)
Child	4.44 (0.656)
	
Relationship to child	
Mother	95.7
Father	2.7
Grandmother	1.6

### Procedures

Parents were recruited to participate in the study during drop off and pick up at the Head Start centers, during parent meetings, and through the registration process at Head Start. Parents were asked if they would like to participate in a study involving three home observations of their dinner meal. The parent who was primarily involved in feeding the Head Start child was designated as the target parent (96% mothers). Staff members from our study explained that the purpose of the study was to better understand what families do during dinner. Consent forms were signed by the target parent at the beginning of the first home observation and confidentiality was assured. During the three home observations, parents and children were observed and coded live during dinner by staff members. Audio/videotapes were also made during the home observations for use as backup for the live coding. Therefore, each of the three home observations included both audio/videotaping of the entire meal and live observation/coding of behavioral feeding practices and domains of the emotional climate of the meal. At the end of each observation, a packet of parent-report questionnaires (available in English and Spanish) was left for the parent to complete. The completed packet was picked up by staff members at the next observation. Parents received an incentive (graduated in amount) at the end of each of the three observations. The study was reviewed and approved by the Institutional Review Board at Baylor College of Medicine.

### Measures

#### Self-reported Feeding Styles

The feeding styles of the parents were measured by the Caregiver's Feeding Styles Questionnaire (CFSQ) [2]. Based on dimensions of demandingness and responsiveness reflective of a general parenting paradigm [7], the CFSQ measures the overall feeding pattern of parents. Dimensions of demandingness and responsiveness are derived through 7 child-centered and 12 parent-centered feeding directives measured on a 5-point Likert scale (ranging from *never *to *always*). *Child-centered feeding *directives are those that promote child autonomy (e.g., reasoning, complimenting, and helping the child to eat). *Parent-centered feeding *are directives that control children's eating through external pressure (e.g., demands, threats, and reward contingencies). A cross classification of high and low scores on the two dimensions indentifies four feeding styles: authoritative (high responsiveness, high demandingness), authoritarian (low responsiveness, high demandingness); indulgent (high responsiveness, low demandingness), and uninvolved (low responsiveness, low demandingness). A more complete discussion of the scoring procedure can be found in a previous study [2]. Convergent validity of the CFSQ has been demonstrated by associations with independent measures of general parenting and authoritarian feeding practices [2]. Differences across feeding styles on an independent measure of children's BMI have been shown in a low-income sample [2]. Evidence of test-retest reliability, internal consistency, convergent validity, and predictive validity has been demonstrated [1-3]. Confirmatory factor analyses support the factorial invariance of this measure across low-income African-American and Hispanic parents [26]. In the current sample, parents were classified into feeding styles as follows: authoritarian (*n *= 35); authoritative (*n *= 55); indulgent (*n *= 51); uninvolved (*n *= 36).

#### Observed Global Emotional Climate

Global emotional climate was measured by the Home Observation Coding System developed by Belsky and colleagues [27] and adapted for the dinner meal. Emotional climate during the meal was defined by five parent behavior domains (variables) that were observed by staff and coded using 5-point global rating scales. The five observed parent domains were positive affect, negative affect, sensitivity, intrusiveness, and detachment. During the duration of each dinner meal, staff members observed and coded the five parent domains in two-minute epochs. At the end of each two-minute epoch, staff members were given one minute to complete global ratings for each of the five domains. The global ratings ranged from 1 (*not-at-all-present*) to 5 (*a-great-deal-present*). Thus, each dinner meal was divided into multiple two-minute epochs of observation followed by one-minute epochs of observer ratings. Parent positive and negative affect were rated separately based on the parent's affect measured by tone, gesture, and facial expressions. Parent sensitivity was rated on the level of sensitivity and responsiveness shown by the parent toward the child. Parent intrusiveness was rated based on the level of insertion of the parent's own agendas with little regard for the child. Parent detachment (which is also considered dismissive) was rated on the level of lack of involvement and responsiveness to the child. At the beginning of each epoch, staff members rated whether there was an opportunity for interaction between the parent and target child. A parent was considered to have an opportunity for interaction if he or she was in visual or verbal proximity of the child. If there was no opportunity for interaction, no ratings were completed for that two-minute epoch. Ratings were composited across the epochs for each individual parent and averaged over the three home observations to construct measures for each of the five parent emotional climate domains (variables).

#### Observed Behavioral Feeding Practices

The 25-item measure used to observe parent behavioral feeding practices was adapted from a previously developed measure (Feeding Behavior Coding System; FBCS) used to observe childcare providers in Head Start centers [28]. Functionally, the FBCS is an observational checklist of the self-report CFSQ. The FBCS documents specific feeding practices exhibited by parents and the frequency of occurrence of practices. Sample practices include reasoning or explaining why a child should eat a food, showing disapproval for not eating, and asking the child to at least try a small bite of the food. Behavioral practice codes were computed by calculating the average frequency for each behavior across the three meals (i.e., frequency/meal).

#### Training

Each staff member in the study who observed during the dinner meals completed extensive training and certification on both of the observational measures (global emotional climate and behavioral feeding practices). Group sessions were conducted with training tapes, so staff members could obtain an in-depth understanding of the constructs to be observed and rated. For the global emotional climate measure, staff were trained to differentiate the frequency and intensity of each of the global rating levels for the five domains. For the behavioral feeding practices, staff were trained to comprehensively understand, differentiate, and rate the 25 behavioral items. Staff members then individually coded "gold standard" audio/video training tapes for each measure and their ratings were compared to the "gold standard." Pilot testing was also conducted with non-study families prior to live coding of study participants to ensure the quality of the procedures and staff member ratings. Periodic re-training was conducted on both measures during the duration of the study to protect against observer drift. Tests of interobserver reliability were conducted before actual data collection and periodically during the study to ensure staff members were consistent in their understanding of the measures and ratings. During the study, reliability checks were conducted for 20% of the global emotional coding observations and 14% of the observed behavioral feeding observations. Correlations between observers for mean emotional climate variables were as follows: positive affect (.66), negative affect (.86), sensitivity (.33), intrusiveness (.82), and detachment (.76). Due to low reliability for the sensitivity variable, this variable was dropped from further analyses.

Live coding of the behavioral feeding practices included 25 variables. Fifteen of the twenty-five variables were dropped due to low base rates (see below) resulting in 10 behavioral feeding practices with sufficient frequency for analysis. Interobserver agreement was calculated by determining for each code whether the two observers agreed (with a margin of two) on its frequency of occurrence for each observation. (Note: because of the high frequency of verbal prompts to eat (M = 25; range = 0 - 131) an agreement was coded if the difference between coders was six or less). Following standard procedures for frequency data, the percent agreement between two observers for the ten feeding behaviors was calculated as follows: helps (100%), spoon feeds/physically intervenes (74%), eat small amount (88%), hurries (90%), reasons (90%), compares (98%), praises/approves (96%), disapproves/scolds (86%), makes positive comments about food (94%) and verbal prompts (74%).

#### Body mass index (BMI)

Height and weight measurements were obtained by trained staff members following procedures described by Lohman, Roche, & Martorell [29]. Parents and children were dressed in light clothing and asked to remove their shoes. Height and weight were measured in duplicate to assure accuracy. Height and weight scores for children were converted to age- and gender- specific BMI z scores using the revised 2000 growth charts from the Centers for Disease Control and Prevention [30]. Height and weight scores for parents were converted to BMI.

### Statistical Analyses

All statistics conducted with the data were run using the Statistical Package for the Social Sciences (SPSS 19.0). Differences across the four feeding styles on emotional climate ratings, behavioral feeding practices, and child BMI were examined through multivariate general linear modeling using MANOVA or MANCOVA. A 2 X 4 MANOVA (ethnicity X feeding style) was run on the emotional climate variables; three MANCOVAs, controlling for meal length, were run on the behavioral feeding practice variables. Because preliminary analyses of the global ratings and feeding practices revealed no significant main effects or interactions involving child gender, child BMI status, or maternal BMI status, these variables were not included as independent variables in these analyses (unfortunately, the sample size prevented examination of the four-way interaction between weight status, gender, ethnicity, and feeding style). To provide a reliable estimate of maternal feeding styles, the global rating and feeding practice variables were averaged across all three meals per family. Estimated marginal means were calculated at the mean meal length (17.5 minutes). A 2 X 2 X 4 MANOVA (ethnicity X gender X feeding style) was run on the child's BMI *z *score (normed for age and gender). Approximate *F *statistics were calculated using Rao's transformation of Wilk's lambda. All significant multivariate effects were followed up by examining the corresponding univariate effects. Significant univariate feeding style effects were followed up with least significant differences post hoc tests. To control for Type 1 error, univariate effects (ANOVAs) were examined only if the corresponding multivariate effects (MANOVA or MANCOVA) were significant at the *p *< .05 level. However, given the exploratory nature of the analyses, the *p *< .10 level was used for all follow-up ANOVAs and post hoc tests [31].

## Results

### Relationship between Feeding Styles and Observed Global Emotional Climate Variables

A 2 X 4 MANOVA (ethnicity X feeding style) was conducted on the four parent emotional climate variables. The MANOVA analysis yielded significant main effects for feeding styles, *F*(12,439) = 3.74, *p *< .001, and ethnicity, *F*(4,166) = 8.85, *p *< .001. Two univariate main effects were significant for ethnicity: positive affect, *F*(1,169) = 35.09, *p *< .001, with Hispanic parents scoring higher (*M *= 2.24, *SD *= .60) than African-American parents (*M *= 1.72*, SD *= .46) and detachment, *F*(1,169) = 4.67, *p *< .05, with African-American parents scoring higher (*M *= 2.20, *SD *= .96) than Hispanic parents (*M *= 1.80, *SD *= .87).

Examination of the univariate analyses for the feeding main effect showed a significant main effect for feeding style for all climate variables except positive affect (see Table 2). Comparison of the four feeding styles on the emotional climate variables (least significant difference post hoc tests) showed numerous differences. As shown in Table 2: 1) parents reporting an authoritarian or uninvolved feeding style exhibited the greatest negative affect; 2) authoritarian parents showed the highest intrusiveness; and 3) indulgent and uninvolved parents showed the greatest detachment. Summarized another way: 1) parents reporting an authoritarian feeding style were high on negative affect and intrusiveness: 2) parents reporting an uninvolved style were high on negative affect and detachment; and 3) authoritative and indulgent parents were low on negative affect and intrusiveness. The only significant difference between the authoritative and indulgent style was that indulgent parents were higher on detachment (although they did not differ from authoritative parents on positive affect).

**Table 2:** Feeding Style Differences on Observed Emotional Climate Variables

	Authoritative	Authoritarian	Indulgent	Uninvolved	*F(3,169)*	*p *<
Positive Affect	2.08	2.12	2.00	1.77	2.37	ns
Negative Affect	1.20_b_	1.50_a_	1.21_b_	1.40_a_	7.44	.001
Intrusiveness	1.52_b_	1.80_a_	1.45_b_	1.38_b_	4.15	.01
Detachment	1.78_b_	1.65_b_	2.17_a_	2.41_a_	5.44	.001

Note: Means with different subscripts were significantly different from one another (*p *< .05).

### Relationship between Feeding Styles and Observed Behavioral Feeding Practices

Before examining group differences on the observed behavioral feeding practices, the total frequencies of occurrence per meal were examined to identify feeding practices that occurred with too low frequency for analysis. Examination of these means identified 15 behavioral feeding practices that occurred an average of less than .5 times per meal: parent takes seconds, enthusiastic modeling, arranging the food, serves, allows the child to choose, offers child second helping (verbal), threatens food punishment, promises food reward, threatens other punishment, promises other reward, negative comments about food, ignores or shows indifference to child, gives child multiple servings of food, allows child to take second or third helping, and offers child second helping (non-verbal) (see Table 3). These low frequency behaviors were not included in the analyses.

**Table 3:** Frequencies of Observed Behavioral Feeding Practices per Meal

	*M*	*SD*	*Ranges*
Modeling			
Parent Takes Seconds	.06	.26	0 to 2
Enthusiastic Modeling	.49	1.10	0 to 6
Situational Management			
Arranging the Food	.11	.33	0 to 2
Serves	.30	.67	0 to 6
Helps	.67	1.16	0 to 6
Allows the Child to Choose	.05	.20	0 to 1
Spoon Feeds/Physically Intervenes	3.32	5.71	0 to 36
Verbal Directives			
Verbal Prompts to Eat	26.26	24.26	0 to 131
Offers Child Second Helping	.21	.49	0 to 3
Tells Child to Eat a Small Amount	.80	1.41	0 to 11
Hurries	1.23	3.52	0 to 31
Reasons	.59	1.41	0 to 14
Comparison	.53	1.66	0 to 14
Threat/Bribe			
Threatens Food Punishment	.26	.75	0 to 5
Promises Food Reward	.29	.67	0 to 5
Threatens Other Punishment	.42	1.14	0 to 10
Promises Other Reward	.27	.63	0 to 4
Scold/Praise			
Praises/Compliments/Approves/Agrees	.54	1.04	0 to 6
Disapproves/Scolds	1.72	3.08	0 to 25
Positive Comments About Food	.52	1.00	0 to 7
Negative Comments About Food	.20	.55	0 to 4
Second Helpings			
Gives Child Multiple Servings of Food	.03	.14	0 to 1
Allows Child to Take 2^nd ^or 3^rd ^Helping	.05	.20	0 to 2
Offers Second Helping (non-verbal)	.05	.16	0 to 1
Other			
Ignores or Shows Indifference to Child	.22	.50	0 to 3

Examination of the feeding practices that occurred with sufficient frequency (see Table 3) showed that verbal prompts to eat were by far the most common followed at a much lower frequency by spoon feeds/physically intervenes and disapproves/scolds. Slightly less frequent than these last two practices were hurries and tells child to eat small amount. These were followed by helps, reasons, praises, comparison, and positive comments about food. Although the average parent made about 26 verbal prompts to eat per meal, the variability was significant, and this variable was positively skewed (skewness = 2.04, SE = .20). For example, 22% of the parents verbally prompted eating an average of 1 to 10 times per meal, 32% prompted 11 to 20 times, 34% prompted 21 to 50 times, and 12% prompted 51 to 131 times. Some of this variation was due to variability in the length of the meals. The length of the meals ranged from 5 to 40 minutes (*M *= 17.68, *SD *= 6.88); however, the correlation between meal length and verbal prompts to eat was only *r*(145) = .27, *p *< .001, and the length of the meals did not differ significantly across feeding styles, *F*(3,132) = 1.39, *p *= .25. Meals for African-American parents were slightly longer (*M *= 18.84, *SD *= 7.14) than meals for Hispanic parents (*M *= 16.69, *SD *= 6.64), *F*(1, 132) = 6.43, *p *< .05. Because most of the behavioral feeding practice variables were positively skewed, the natural log transformation was applied to each variable before the analyses. Also, the length of the meal was controlled for through the use of multivariate analyses of covariance.

Three 2 X 4 MANCOVAs (ethnicity X feeding style) were conducted on the observed behavioral feeding practices: one for the two situational management codes (helps and spoon feeds/physically intervenes); one for the five verbal directive codes (verbal prompts to eat, tells child to eat a small amount, hurries, reasons, and comparison); and one for the three scold/praise codes (praises, disapproves/scolds, and positive comments about food). The ethnicity main effect was significant for both the situational management, *F*(2, 130) = 11.87, *p *< .001, and the verbal directives, *F*(5, 127) = 5.42, *p *< .001, analyses. The feeding style main effect was significant in all three analyses: situational management, *F*(6, 260) = 2.26, *p *< .05; verbal directives, *F*(15, 361) = 1.79, *p *< .05; and scold/praise, *F*(9, 314) = 2.83, *p *< .01.

Univariate analyses of variance revealed significant effects of ethnicity for all of the practices (*p *< .01) in the situational management and verbal directives analyses. In every case, Hispanic parents engaged in all of the practices more frequently than did African-American parents. The univariate analyses for feeding style revealed significant effects for four practices (i.e., spoon feeds/physically intervenes, eat small amount, hurries, and disapproves/scolds) and trends (*p *< .10) for three others (i.e., verbal prompts to eat, reasons and positive comments about food). Least significant difference post hoc tests were used to examine differences between the feeding styles (see Table 4). The significant mean differences revealed in the least significant differences tests (all *p*s < .05) can be described as follows: 1) parents with feeding styles characterized by high demands (authoritative and authoritarian) were most likely to use spoon feeding/physical intervention, to verbally prompt the child to eat, to use reasoning, and to make positive comments about food; and 2) authoritarian parents were more likely to try to get the child to eat a small amount, to hurry eating, and to disapprove of or scold the child. Parents with styles characterized by low demands (indulgent and uninvolved) were significantly lower on most of the behavioral feeding practices compared to the high demand parents and did not significantly differ from one another.

**Table 4:** Feeding Style Differences on Observed Behavioral Feeding Practices (Natural Log Transformed)

	Authoritative	Authoritarian	Indulgent	Uninvolved	*F(3,131)*	*p *<
Situational Management						
Helps	.26	.39	.34	.23	.94	ns
Spoon Feeds/Physically Intervenes	.81_ab_	1.22_a_	.65_b_	.67_ab_	3.81	.05
Verbal Directives						
Verbal Prompts to Eat	2.93_ab_	3.12_a_	2.76_b_	2.81_b_	2.18	.10
Eat Small Amount	.17_b_	.61_a_	.38_b_	.33_b_	3.94	.01
Hurries	.18_b_	.58_a_	.23_b_	.29_b_	3.12	.05
Reasons	.25_ab_	.44_a_	.19_b_	.26_ab_	2.51	.07
Comparison	.16	.25	.16	.25	.38	ns
Scold/Praise						
Praises/Approves	.39	.38	.16	.22	2.04	ns
Disapproves/Scolds	.58_b_	1.07_a_	.49_b_	.73_b_	5.84	.001
Positive Comments Food	.30_ab_	.43_a_	.28_ab_	.15_b_	2.53	.06

Note: Estimated marginal means were calculated for the feeding practices at the mean meal length (17.5 minutes). Means with different subscripts were significantly different from one another (*p *< .05)

### Relationship between Self-Reported Parental Feeding Styles and Child BMI

A 2 X 2 X 4 MANOVA (ethnicity X gender X feeding style) was run on the children's BMI *z *scores (normed for age and gender). Only the ethnicity by gender by feeding style interaction was significant, *F*(3,157) = 3.20, *p *< .05. This interaction was followed up with four simple main effects analyses examining the effects of feeding style (one for each ethnicity by gender combination). Of these, only one group showed a main effect for feeding style--Hispanic boys, *F*(3,48) = 4.98, *p *< .01. Least significant difference post hoc tests showed that the mean child BMI *z *score was significantly greater (p < .05) for Hispanic boys of indulgent parents (*M *= 2.14, *SD *= 1.23) than for Hispanic boys in the other three groups (authoritative: *M *= -.16, *SD *= 1.04; authoritarian: *M *= .70, *SD *= 1.47; uninvolved: *M *= .26, *SD *= 1.66). The other three groups did not significantly differ from one another.

## Discussion

The overall aim of this study was to observe differences in the emotional climate created by parents during dinner and specific behavioral feeding practices that may interfere with child self-regulation among those reporting different feeding styles on the CFSQ. This was done in order to better understand the consistent relationship found in previous studies between self-reports of an indulgent parenting and/or feeding style and higher child weight. Cross-sectional [1-3] and longitudinal [4] studies of ethnically diverse, low-income parents have found that young children of indulgent parents were most likely to have a higher weight status or become overweight when measured three years later. Indulgent feeding has also been linked to child eating behaviors among diverse ethnic groups [5], Hennessy E, Hughes SO, Goldberg JP, Hyatt RR, Economos CD: Permissive parental feeding behavior is associated with an increase in low nutrient-dense foods among American children living in rural communities, submitted].

In an attempt to better understand mechanisms that might help explain this association between indulgence and child weight, we were specifically interested in *how *parents interacted with their children during the dinner meal. We observed that parents with an indulgent feeding style were significantly different from other feeding styles on three of the four emotional climate variables. Additionally, indulgent parents were observed to be significantly lower on *almost all *of the observed behavioral feeding practices. Our results confirmed that parents with self-reported indulgent feeding styles made *very few *demands on their children to eat during dinner and were less *negative *and *intrusive *with their children during the meal. Surprisingly, these parents also showed higher *emotional detachment *with their children during dinner. Because detachment was defined as low levels of involvement and responsiveness toward the child, this finding may have been a consequence of the raters' observation of low parental involvement during meals. Because styles of parenting/feeding constitute ways that parents establish and manage the home environment, it is expected that indulgent parents are trying to control the emotional climate of the meal by making sure their child is happy. Indulgent parents are supportive and non-directive with their child but do not spend a lot of time at the task of getting their child to eat.

The current data also replicate findings of previous research showing that children of parents with an indulgent feeding style are at the greatest risk for obesity [1-4]. However, in the current study, this finding was significant *only *for Hispanic boys. This finding is unexpected given that the families in this study were similar demographically to the families in our previous research [2-4]. Because Hispanic preschool boys show higher obesity rates than Hispanic girls or African-American boys or girls [32], it appears that the indulgent feeding style only had effects on child weight status for this high-risk demographic group.

The association between indulgent feeding style and child obesity suggests that too little control may be just as problematic for children as too much control [cf., [21]]. If parents do not provide enough emotional investment and supervision in the eating context (essentially allowing children to consume as much energy-dense food as they wish), their children may ignore their internal fullness cues resulting in inappropriate weight gain. An intermediate level of parental involvement may be optimal. This is supported by research by Jansen and colleagues [33] who found that both *high *and *low *levels of parental restrictiveness in feeding were related to the amount of snacks that children consumed in their parents' absence in a laboratory setting--i.e., intermediate levels of parental restriction were associated with the lowest levels of snack consumption. Such results are consistent with the predictions of self-determination theory, which posits that intrinsic motivation is facilitated by autonomy supporting practices [34,35]. As demonstrated in research in other domains, young children with high levels of self-regulation have parents who provide them with sufficient attention, assistance, and support to complete difficult tasks that they cannot complete on their own (see Power, 2004 for a review) [10]. The same may hold true for children in the eating context.

Our analyses revealed other significant feeding style differences in the emotional climate of the meal and in the behavioral feeding practices that parents used to get their children to eat. As expected, authoritarian parents exhibited significantly higher negative affect and intrusiveness during the meal compared to the authoritative and indulgent parents. Uninvolved parents, also as hypothesized, exhibited higher detachment during dinner compared to authoritative and authoritarian parents. The uninvolved parents also exhibited higher negative affect relative to authoritative and indulgent parents. Even though parents with an indulgent style showed lower negative affect, as expected, they were also observed to exhibit more detachment similar to the uninvolved parents.

Most of the differences between the self-reported feeding styles on the behavioral feeding practices variables were seen between the authoritarian parents and the indulgent parents. Parents with an authoritarian feeding style were significantly higher on *all *of the observed feeding practices except for making positive comments about food compared to parents with an indulgent style. Furthermore, parents reporting authoritarian and authoritative feeding styles (high demandingness) used spoon feeding/physical interventions and verbal prompts to eat more frequently than parents with indulgent and uninvolved styles (low demandingness). Parents with authoritarian and authoritative styles also showed more reasoning and made more positive comments about food than parents with styles characterized by low demandingness. Parents with authoritarian feeding styles engaged in practices such as disapproving of and scolding their child, hurrying their child, and asking their child to eat a small amount more frequently than did parents in the other three groups.

This study extends our understanding of the relationship between feeding styles, the emotional climate of the meal, and feeding behaviors/practices of parents [1-3]. Results suggest that the emotional climate of the dinner meal may play an important part in how parents socialize their children around eating. Results also suggest that parents' self-reported feeding styles might be a proxy for the emotional climate of the dinner meal, which may in turn influence the child's eating behaviors and weight status. Parental behaviors exhibited during the child's eating activities and the emotional climate created by these behaviors can significantly impact the eating behaviors of the developing child [36] in a positive or a negative way depending upon the feeding style of the parent [37-40].

One of the most significant strengths of this study was the use of direct observation to examine parent-child interactions during home meals. Direct observation plays a significant role in advancing our understanding of the family process during mealtime. By observing family dynamics during dinner as opposed to relying on self-report data, researchers can draw conclusions about parent-child interactions that may be difficult to assess through self-report [41]. For example, parents may underreport feeding practices such as ignoring the child, yelling at the child, or forcing the child to eat. Some parents may also inaccurately self-report their behaviors due to cultural norms, language barriers, or lack of awareness [42]. Observational data also provide more detailed information about the how and why of the parent-child dynamic thus offering a link between qualitative data and quantitative research methods. One limitation of being observed in the home is that observation may impact parents' usual meal time practices. The problem of participant reactivity has been addressed in a review article by Gardner [43]. The author suggests that the presence of an observer does not markedly distort participant behaviors. She also found no differences in the frequency and nature of behaviors between the first and later observations and little evidence of systematic changes in the frequency of negative and positive behaviors.

While one strength of this study was multiple observations on each family, these observations were conducted in a relatively short time frame such that seasonal influences on food intake could have been present. It is also possible that by observing the family three times during the two- to three-week interval and only at the dinner meal did not allow for the entire spectrum of parental feeding behaviors to be observed. Furthermore, as noted by other researchers, parents adapt their feeding practices to the child's traits, needs, and weight [44]. For example, if the child has recently gained weight at the time of the observation, the parent might discourage certain types of food (low-nutrient energy-dense foods). As children's weight sometimes fluctuates when they are young, a snapshot of feeding such as the one collected during this study may not be representative of usual parent behavior. A longitudinal study, over the course of a year or two, could resolve this issue.

This work suggests that feeding styles play a crucial role in the emotional climate of the dinner meal. Furthermore, the emotional climate created by the parents and the specific feeding practices used to get children to eat may negatively influence the child's internal cues of fullness and self-regulation. This learned process can carry over into the child's adult life. An authoritarian parent might be able to "control" the child's weight at a healthy level while the child is very young, but if the child does not learn to self-regulate eating during childhood, he/she might gain weight when no longer under the parent's complete control (e.g., when the child enters elementary school and has access to other foods beyond the parents' control). The information from this study may be used to improve family interactions around eating while the child is still young. Interventions to improve parent-child interactions during meal time could focus on getting indulgent parents to make more demands on their child to eat and setting boundaries regarding what types of food should be eaten. Furthermore, authoritarian parents could be taught to use less controlling practices such as physical interventions, hurrying, and disapproving of or scolding their child. These interventions may help children to learn to have a good relationship with food.

## Competing interests

The authors declare that they have no competing interests.

## Authors' contributions

SOH, TGP, MAP, MBC, TAN, SKH, RMS made substantial contributions to the conception, design, acquisition, analysis and interpretation of the data. SOH, TGP, MAP, MBC, RMS have been involved in drafting the manuscript or revising it critically for important intellectual content. SOH, TGP, MAP, MBC, TAN, SKH, RMS have given final approval of the version to be published.
